# Supplementary material for: Targeted single cell expression profiling identifies integrators of sleep and metabolic state
Source: G3 (Bethesda). 2025 Jun 13;15(7):jkaf079. doi: 10.1093/g3journal/jkaf079 (PMC12239624; doi:10.1093/g3journal/jkaf079)
Supplement: jkaf079_Supplementary_Data [file jkaf079_supplementary_data.pdf]

CEL-Seq2 protocol adapted for GFP-labeled neurons in flies

Reagents

comsumable	brand	cat#
LoBind 0.5 mL tubes	Eppendorf	22431005
UltraPure™ DNase/RNase-Free Distilled Water	ThermoFisher Scientific	10977023
Ethanol		
Bioanalyzer High Sensitivity DNA kits	Agilent	5067-4626
Bioanalyzer RNA pico kits	Agilent	5067-1513
ERCC RNA spike-in mix	ThermoFisher Scientific	4456740
dNTPs mix		
SuperScript II	ThermoFisher Scientific	18064014
RNasin® Plus RNase Inhibitor	Promega	N2615
Second strand buffer	ThermoFisher Scientific	10812014
DNA Polymerase I (E. coli)	ThermoFisher Scientific	18010025
E. coli DNA ligase	ThermoFisher Scientific	18052019
RnaseH (E. coli)	ThermoFisher Scientific	18021071
MEGAscript T7 Transcription Kit	ThermoFisher Scientific	AM1334
ExoSAP-IT	ThermoFisher Scientific	78200.200.UL
Fragmentation stop buffer: EDTA (0.5 M), pH 8.0	ThermoFisher Scientific	AM9260G
NEBNext Magnesium RNA Fragmentation Module	New England BioLabs	E6150S
Trizma acetate	Sigma-Aldrich	T1258-100G
Potassium acetate solution	Sigma-Aldrich	95843-100ML-F
Magnesium acetate solution	Sigma-Aldrich	63052-100ML
Polyethylene glycol 8000	Sigma-Aldrich	P5413-500G
AMPure XP beads	Beckman Coulter	A63880
RNAClean XP beads	Beckman Coulter	A63987
Dental wax		
DynaMag™-2 Magnet	ThermoFisher Scientific	12321D

comsumable	brand	cat#
Phusion® High-Fidelity PCR Master Mix with HF Buffer	New England BioLabs	M0531S
randomhexRT primer	IDT	GCCTTGGCACCCGAGAATTCCANNNNNN
RNA PCR Primer (RP1)	Illumina	AATGATACGGCGACCACCGAGATCTACACGTT CAGAGTTCTACAGTCCGA
RNA PCR Index Primers RPI1	Illumina	CAAGCAGAAGACGGCATACGAGATCGTGATGT GACTGGAGTTCCTTGGCACCCGAGAATTCCA
RNA PCR Index Primers RPI3		CAAGCAGAAGACGGCATACGAGATGCCTAAGT GACTGGAGTTCCTTGGCACCCGAGAATTCCA
RNA PCR Index Primers RPI7		CAAGCAGAAGACGGCATACGAGATGATCTGGT GACTGGAGTTCCTTGGCACCCGAGAATTCCA
RNA PCR Index Primers RPI2	Illumina	CAAGCAGAAGACGGCATACGAGATACATCGGT GACTGGAGTTCCTTGGCACCCGAGAATTCCA
RNA PCR Index Primers RPI4		CAAGCAGAAGACGGCATACGAGATTGGTCAGT GACTGGAGTTCCTTGGCACCCGAGAATTCCA
RNA PCR Index Primers RPI8		CAAGCAGAAGACGGCATACGAGATTCAAGTGT GACTGGAGTTCCTTGGCACCCGAGAATTCCA
RNA PCR Index Primers RPI6	Illumina	CAAGCAGAAGACGGCATACGAGATATTGGCGT GACTGGAGTTCCTTGGCACCCGAGAATTCCA
RNA PCR Index Primers RPI10		CAAGCAGAAGACGGCATACGAGATAAGCTAGT GACTGGAGTTCCTTGGCACCCGAGAATTCCA
RNA PCR Index Primers RPI12		CAAGCAGAAGACGGCATACGAGATTACAAGGT GACTGGAGTTCCTTGGCACCCGAGAATTCCA

CEL-Seq2 Primers (ThermoFisher)

1s	GCCGGTAATACGACTCACTATAGGGAGTTCTACAGTCCGACGATCNNNNNNAGACTCTTTTTTTTTTTTTTTTTTTTTTTTV
4s	GCCGGTAATACGACTCACTATAGGGAGTTCTACAGTCCGACGATCNNNNNNAGCTTCTTTTTTTTTTTTTTTTTTTTTTTTV
5s	GCCGGTAATACGACTCACTATAGGGAGTTCTACAGTCCGACGATCNNNNNNCATGAGTTTTTTTTTTTTTTTTTTTTTTTTV
9s	GCCGGTAATACGACTCACTATAGGGAGTTCTACAGTCCGACGATCNNNNNNCAGATCTTTTTTTTTTTTTTTTTTTTTTTTV
10s	GCCGGTAATACGACTCACTATAGGGAGTTCTACAGTCCGACGATCNNNNNNTCACAGTTTTTTTTTTTTTTTTTTTTTTTTV
23s	GCCGGTAATACGACTCACTATAGGGAGTTCTACAGTCCGACGATCNNNNNNGTCTAGTTTTTTTTTTTTTTTTTTTTTTTTV
25s	GCCGGTAATACGACTCACTATAGGGAGTTCTACAGTCCGACGATCNNNNNNGTTGCATTTTTTTTTTTTTTTTTTTTTTTTV
26s	GCCGGTAATACGACTCACTATAGGGAGTTCTACAGTCCGACGATCNNNNNNGTGACATTTTTTTTTTTTTTTTTTTTTTTTV
31s	GCCGGTAATACGACTCACTATAGGGAGTTCTACAGTCCGACGATCNNNNNNACTCGATTTTTTTTTTTTTTTTTTTTTTTTV
46s	GCCGGTAATACGACTCACTATAGGGAGTTCTACAGTCCGACGATCNNNNNNTGCAGATTTTTTTTTTTTTTTTTTTTTTTTV
6s	GCCGGTAATACGACTCACTATAGGGAGTTCTACAGTCCGACGATCNNNNNNCATGCATTTTTTTTTTTTTTTTTTTTTTTTV
14s	GCCGGTAATACGACTCACTATAGGGAGTTCTACAGTCCGACGATCNNNNNNTCCTAGTTTTTTTTTTTTTTTTTTTTTTTTV
20s	GCCGGTAATACGACTCACTATAGGGAGTTCTACAGTCCGACGATCNNNNNNGTACAGTTTTTTTTTTTTTTTTTTTTTTTTV
28s	GCCGGTAATACGACTCACTATAGGGAGTTCTACAGTCCGACGATCNNNNNNACAGTGTTTTTTTTTTTTTTTTTTTTTTTTV
35s	GCCGGTAATACGACTCACTATAGGGAGTTCTACAGTCCGACGATCNNNNNNCTAGACTTTTTTTTTTTTTTTTTTTTTTTTV
43s	GCCGGTAATACGACTCACTATAGGGAGTTCTACAGTCCGACGATCNNNNNNTGAGACTTTTTTTTTTTTTTTTTTTTTTTTV
2s	GCCGGTAATACGACTCACTATAGGGAGTTCTACAGTCCGACGATCNNNNNNAGCTAGTTTTTTTTTTTTTTTTTTTTTTTTV
17s	GCCGGTAATACGACTCACTATAGGGAGTTCTACAGTCCGACGATCNNNNNNTCGAAGTTTTTTTTTTTTTTTTTTTTTTTTV
27s	GCCGGTAATACGACTCACTATAGGGAGTTCTACAGTCCGACGATCNNNNNNGTGATCTTTTTTTTTTTTTTTTTTTTTTTTV
42s	GCCGGTAATACGACTCACTATAGGGAGTTCTACAGTCCGACGATCNNNNNNCTGTGATTTTTTTTTTTTTTTTTTTTTTTTV

Pipette solution

Reagent	volume (µL)	
Filtered PBS	190	
RNasin Plus (40 U/µL)	10	
total	200	

Tube solution

Reagent	volume (µL)	
broken tip with cell (PBS w/ RNasin Plus)	1.4	
H_2O	1.0	
RNasin Plus (40 U/µL)	0.35	
total	1.35	

CS mix1

Reagent	volume (µL)	
Primer (25 ng/µL)	0.5	
ERCC Spike-in 1:400,000 dilution	0.4	Crosses 5 orders of molecules in each single cell
dNTPs (10 mM)	0.3	
total	1.2	

CS mix2

Reagent	volume (µL)	X21
First Strand Buffer	1.2	25.2
100 mM DTT	0.6	12.6
SS II	0.3	6.3
total	2.1	

Bead binding buffer (20% PEG8000, 2.5M NaCl)

Reagent	volume (µL)	
40% PEG8000 stock	500	Dissolve PEG 8000 in pure H_2O. **Pass it through a 0.22-µm filter**. Store the solution at room temperature.
5M NaCl	500	
total	1000	

Single cell harvesting & RNA amplification

Harvesting single cells

1. Prepare solutions and pipettes (#2 to make pipettes for DPM of 12-μm ⌀; #5 for LHLK of 8-μm ⌀)
2. Fill the 0.5 mL LoBind tubes with 1.35 μL of Tube solution and number
3. Prepare liquid nitrogen
4. Glue the flies on the Fly Stage, two flies per batch; while waiting the glue dry, clean two previously used Fly Stages
5. Dissect a fly. Making sure the BBB removed by forceps/tweezers
6. Harvest the target cell with several gentle suction pulses
7. Plug the pipette with dental wax
8. Break the very end of pipette tip against the bottom of tube, immediately mix and spin down, and then throw the tube into liquid nitrogen
   # Stopping point — the tubes can be stored at -80 ℃

Annealing with primer

9. Match the cell# to a compatible primer set.
10. Prepare at least 4 replicates of CS mix1 for each primer (for more precise pipetting)
11. Take the tubes out of -80 ℃ with dewar and spin down at maximum speed for 30 sec
12. Add 1.2 μL of CS mix1
13. Incubate the tubes at 65 ℃ for 5 min (with lid of thermal cycler set to 65 ℃)
14. Immediately cool on ice for 1 min, briefly centrifuge at maximal speed for 30 sec to bring the contents to the tube bottom, and then return to ice.

RT reaction

15. Add 2.1 μL of CS mix2 and incubate at 42 ℃ for 1 hr with 50 ℃ lid. Total volume should be 6 μL.
16. Heat-inactivate the reverse transcriptase by incubating at 70 ℃ for 10 min.

Second strand reaction

17. Move previous step to ice so it cools below 16 ℃
18. Add 18 μL of the following mix to each reaction tube:

Reagent	volume (μL)	X21
H_2O	12.13	254.73
Second strand buffer	4.5	94.5
dNTP	0.45	9.45
Ligase	0.16	3.36
E. coli DNA polymerase	0.6	12.6
RNase H	0.16	3.36
total	18	

   Flick and spin samples at maximal speed for a few seconds (**No vortexing/pipetting**)

19. Incubate at 16 ℃ for 2 hr in Veriti thermal cycler with unheated lid

cDNA cleanup

20. Prewarm AMPure XP beads to room temperature
21. Pool all cells that are to go to same IVT. Should have ~22 μL from each cell
22. Vortex AMPure XP Beads until well dispersed, then add to pooled sample (< 120 μL/ tube) 0.2X beads and 1X bead binding buffer.
    # 120/110/100 sample - 24/22/20 beads - 120/110/100 BBB

23. Incubate at R.T. for 15 min
24. Place on magnetic stand for at least 5 min, until liquid appears clear
25. Remove and discard 200 μL of the supernatant
26. Add 190 μL freshly prepared 80% EtOH
27. Incubate at least 30 sec., then remove and discard supernatant without disturbing beads
28. Repeat 25 - 26
29. Air dry beads for 15 min until completely dry
30. Resuspend **and combine** with 6.4 μL water. Pipette the entire volume up and down ten times to mix thoroughly
31. Incubate at R.T. for 2 min
32. Go straight to IVT with beads

IVT

33. Prepare the following mix and add 9.6 μL per tube (scale up if cDNA volume is more than 6.4)
    # 10X T7 buffer should be kept at R.T.
    # Assemble the reaction at R.T. to avoid precipitation of template DNA

A	1.6 μL
G	1.6 μL
C	1.6 μL
U	1.6 μL
10X T7 buffer	1.6 μL
T7 enzyme	1.6 μL

34. Incubate in a thermal cycler at 37 ℃ for 13 hrs, with lid at 70 ℃. Set cycler to go to 4 ℃ at end of incubation. aRNA (amplified RNA) is stable for at least several hours.

EXO-SAP treatment (to remove primers)

35. Add 6 μL enzyme (ExoSAP-IT, Affymetrix 78200)
36. Incubate at 37 ℃ for 30 min.

RNA fragmentation

37. Mix the following on ice:

aRNA	22 μL
NEB RNA fragmentation buffer	5.5 μL

38. Incubate at 94 ℃ for 2 min
39. Immediately move to ice and add 2.75 μL fragmentation stop buffer

Remove beads

40. Place on magnetic stand for at least 5 min, until liquid appears clear
41. Transfer the supernatant to new tube

aRNA cleanup

42. Pre-warm RNAClean XP beads to R.T.
43. Vortex RNAClean XP beads until well dispersed, add to sample 55 µL beads. (1.8 volumes)
44. Incubate at R.T. for 10 min
45. Place on magnetic stand for at least 5 min, until liquid appears clear
46. Remove and discard ~80 µL of the supernatant
47. Add 190 µL freshly prepared 70% EtOH
48. Incubate at least 30 sec., then remove and discard supernatant without disturbing beads
49. Repeat wash two more times
50. Air dry beads for 15 min, or until completely dry
51. Resuspend with 7 µL water. Pipette entire volume up and down ten times to mix thoroughly
52. Incubate at R.T. for 2 min
53. Place on magnetics stand for 5 min, until liquid appears clear
54. Transfer supernatant to a new tube

 # Stopping point — the tubes can be stored at -80 ℃

 # When starting with few or very small cells aRNA is not always visible, but this is not necessarily an indication of failed amplification. Go straight to library preparation, resuspend beads above in 5.5 µL instead of 7 µL.

Check aRNA amount and quality

55. Load 1 µL onto Bioanalyzer RNA pico chip (after heating an aliquot of the sample to 70 ℃ for 2 min).
    # When starting the IVT with ~0.1 ng total RNA, the expected yield is 500-1000 pg/µL. Size distribution should peak at ~500 bp

Library preparation

RT reaction

56. To 5 µL RNA add 1 µL randomhexRT primer and 0.5 µL dNTPs
57. Incubate at 65 ℃ for 5 min
58. Quick chill on ice
59. Add 4 µL of the following mix at R.T. to each reaction

First strand buffer	2 µL
DTT (0.1M)	1 µL
RNasin Plus	0.5 µL
Superscript II	0.5 µL

60. Incubate at 25 ℃ for 10 min
61. Incubate at 42 ℃ for 1 hr with lid at 50 ℃

PCR amplification

62. To each reverse transcription reaction add 38 µL of the following mix:

Water	11 µL
PCR mix	25 µL
(Phusion High-Fidelity PCR Master Mix with HF Buffer (NEB M0531)	
RNA PCR Primer (RP1, from Illumina kit)	2 µL
RNA PCR Index# (RPI#)	2 µL

 # For RPI#, choose balanced primers according to Illumina's pooling guide

e.g. RPI1, 3, and 7 / RPI2, 4, and 8/ RPI6, 10, and 12.

63. Amplify the tube in the thermal cycler using the following PCR cycling conditions
    - 30 sec. at 98 ℃
    - 11 cycles of
        - 10 sec. at 98 ℃
        - 30 sec. at 60 ℃
        - 30 sec. at 72 ℃
    - 10 min. at 72 ℃
    - Hold at 4 ℃

    # Can go up to 15 cycles if necessary, if aRNA concentration was low

    # Stopping point — the tubes can be stored at -20 ℃

Bead cleanup of PCR products — Repeat 1

64. Pre-warm beads to R.T.
65. Vortex AMPure XP beads until well dispersed, then add 50 µL to the 50 µL PCR reaction (or 25 µL if half a PCR reaction was performed). Mix entire volume up ten times to mix thoroughly
66. Incubate at R.T. for 15 min
67. Place on magnetic stand for at least 5 min, until liquid appears clear
68. Remove and discard 95 µL (or 45 µL) of the supernatant
69. Add 200 µL freshly prepared 80% EtOH
70. Incubate at least 30 sec., then remove and discard supernatant without disturbing beads
71. Repeat 67 - 68
72. Air dry beads for 15 min, or until completely dry
73. Resuspend with 25 µL water. Pipette entire volume up and down ten times to mix thoroughly
74. Incubate at R.T. for 2 min
75. Place on magnetic stand for 5 min, until liquid appears clear
76. Transfer 25 µL of supernatant to a new tube

Bead cleanup of PCR products — Repeat 2

77. Repeat as above, adding 25 µL beads and eluting in 10 µL water at the end, transferring 10 µL to a new tube

Check library amount and quality

78. (Optional?) Check concentration of DNA by Qubit, 1µL should be enough to measure using the high sensitivity reagent; expected concentration is at least ~1 ng/µL
79. Run 1 µL of each sample on Bioanalyzer using a high sensitivity DNA chip to see size distribution. Expected peak at 200 - 400 bp

Sequencing

80. Prepare 10 nM in 20 µL for CSHL Genome Center, Woodbury, to run HiSeq2500 RAPID v.3 reagents, PE25X50 (7 for the illumine index when needed).

Reference:

Hashimshony, T., Hashimshony, T., Wagner, F., Wagner, F., Sher, N., Sher, N., et al. (2012). CEL-Seq: single-cell RNA-Seq by multiplexed linear amplification. Cell Reports, 2(3), 666–673. http://doi.org/10.1016/j.celrep.2012.08.003

Hashimshony, T., Senderovich, N., Avital, G., Klochendler, A., de Leeuw, Y., Anavy, L., et al. (2016). CEL-Seq2: sensitive highly-multiplexed single-cell RNA-Seq. Genome Biology, 17(1), 77. http://doi.org/10.1186/s13059-016-0938-8
